# Barriers and facilitators of cancer genetic risk screening at community-based organizations serving Latinas

**DOI:** 10.1007/s12687-025-00839-7

**Published:** 2025-12-16

**Authors:** Bella Ortega, Maisha R. Huq, Dariana Sedeño-Delgado, Clara Barajas, Sara Gómez-Trillos, Geoffrey Curran, Kristi D. Graves, Vanessa B. Sheppard, Marc D. Schwartz, Beth N. Peshkin, Claudia Campos, Nancy Valencia-Rojas, Gina Hernández, Nathaly Garces, Chiranjeev Dash, Suzanne O’Neill, Laura A. Logie, Astrid Jimenez, Mary Mills, Brenda V. Roig, Antonio Villa, Kennya Alvarado, Paula Cupertino, Pilar Carrera, Alejandra Hurtado-de-Mendoza

**Affiliations:** 1 Department of Oncology, Georgetown University Medical Center, Washington DC, USA; 2 Jess and Mildred Fisher Center for Hereditary Cancer and Clinical Cancer Genomics Research, Georgetown University, Washington DC, USA; 3 Department of Pharmacy Practice, College of Pharmacy, University of Arkansas for Medical Sciences, Little Rock, AR USA; 4 School of Public Health, Department of Social and Behavioral Sciences, Virginia Commonwealth University, Richmond, VA USA; 5 Nueva Vida, Inc., Alexandria, VA USA; 6 La Casa de la Salud, Midlothian, VA USA; 7 Arlington Free Clinic, Arlington, VA USA; 8 Department of Surgery, University of Rochester Medical Center, Rochester, NY USA; 9 Universidad Autónoma de Madrid, Madrid, Spain; 10 Present Address: Georgetown Lombardi Comprehensive Cancer Center, Georgetown University, Wisconsin Ave, Washington DC, 20007 USA

**Keywords:** Community-based organizations, Cancer genetic risk screening, Latina, Health equity, Adaptations

## Abstract

Cancer genetic counseling and testing is potentially lifesaving for individuals at risk of hereditary cancers. Yet, it is severely underutilized among under-resourced Latinas. There has been limited examination of implementing cancer genetic risk screening—the first step of cancer genetic counseling and testing –via community-based organizations (CBOs) serving Latinas. This project explored multilevel barriers and facilitators to implementing cancer genetic risk screening among four CBOs serving Latinas in the Washington DC-Virginia area. We conducted four focus groups with 26 staff at CBOs that implemented a genetic risk screener from January-September 2021. We employed template analysis and a modified Consolidated Framework for Implementation Research (CFIR)—consisting of CFIR’s original five domains (*Process*,* Intervention*,* Inner Setting*,* Outer Setting*,* Characteristics of Individuals*) and CFIR 2.0’s Adapting construct and a lens emphasizing Health Equity across all domains — to identify barriers and facilitators to implementation. CBOs administered the risk screener to 789 Latinas. A prominent *Process* barrier was not having the optimal data management system for screening. CBO staff preferred the *Intervention* (i.e. the screener) over previous family history data collection. Adapting the screener to organizational infrastructure and patient-level health literacy and cultural responsiveness barriers were facilitators. Lack of *Inner Setting* staff time to conduct screening was a barrier. Stronger systems of CBOs’ *Outer Setting* partnerships facilitated screening. *Characteristics of Individuals* that promoted GCT screening was CBO staff knowledge, beliefs, and self-efficacy towards risk screening. Provided that key barriers are addressed and facilitators are leveraged during implementation, genetic risk screening may potentially be feasible and acceptable even across heterogeneous CBOs. Future research evaluating feasibility and acceptability of genetic risk screening in CBOs is needed.

**Supplementary Information:**

The online version contains supplementary material available at 10.1007/s12687-025-00839-7.

## Background

Guidelines from the National Comprehensive Cancer Network (NCCN) recommend cancer genetic counseling and testing (GCT) for individuals at increased risk of hereditary breast and ovarian cancer (HBOC) (Daly et al. 2021). GCT-based risk management strategies can reduce breast and ovarian cancer mortality by > 70% and risk by 70–90% among women carrying inherited gene mutations (i.e. pathogenic variants; PVs) (Salhab and Bismohun, 2010; Liu et al. 2023; Bernstein et al. 2015; Prince et al. 2017; De Jong et al. 2006; Valachis et al. 2014). Because HBOC substantially increases lifetime cancer risk—with female BRCA1/BRCA2 PV carriers facing up to six times the risk of breast cancer and 40 times the risk of ovarian cancer compared to non-carriers (Garutti et al. 2023; Imyanitov et al. 2023)—the health benefits of GCT are potentially lifesaving.

However, Latinas are significantly less likely to access GCT compared to non-Hispanic White women (Dean et al. 2015; Cragun et al. 2015; Levy et al. 2011; Armstrong 2005). This disparity reflects broader patterns of underutilization across minoritized groups (Tawfik et al. 2023; Clarke and Van El 2022; Parikh et al. 2020; Paiella et al. 2023; Mittendorf et al. 2021; Suther and Gebre-Egziabher 2009; Sayani 2019; Chapman-Davis et al. 2021; Dharwadkar et al. 2022; Muller et al. 2018). Latinas face a range of systemic and psychosocial barriers that contribute to GCT inequities. Structural barriers include lack of insurance or underinsurance and high cost (Hurtado-de-Mendoza et al. 2018; Dron et al. 2023), limited access to culturally and linguistically appropriate services (Augusto et al. 2019), lack of provider-initiated communication about and referrals for GCT (Chavez-Yenter et al. 2021; Hurtado-de-Mendoza et al. 2018), difficulty navigating complex healthcare systems not designed for the needs of immigrant or underserved communities (Augusto et al. 2019; Southwick et al. 2020), and medical mistrust (Hann et al. 2017). Psychosocial barriers include low familiarity with genetic testing (Gómez‐Trillos et al. 2020), limited awareness of family health history (Hurtado-de-Mendoza et al. 2018; Ricker et al. 2018; Gómez‐Trillos et al.^2020^), and cultural stigmas surrounding cancer (Rajpal et al. 2017).

The first step of the GCT continuum of services is genetic risk screening to identify high-risk individuals. For genetic risk screening, the United States Preventive Services Task Force (USPSTF) recommends five brief validated familial cancer history screening tools (Ontario Family History Assessment Tool, Manchester Scoring System, Referral Screening Tool, Pedigree Assessment Tool, and the Family History Screening; FHS-7) (Moyer 2014). Yet, at the health system level, the lack of routine HBOC risk screening serves as a health system-level barrier contributing to lower GCT access and suboptimal referrals (Olufosoye et al. 2024; Williams et al. 2019; Hurtado-de-Mendoza et al. 2018; Chavez-Yenter et al. 2025) among Latinas.

Community-based organizations (CBOs) are uniquely positioned to help close the gap in genetic risk screening and GCT access among Latinas (Almeida et al. 2021; Perez et al. 2023; Vadaparampil et al. 2022). CBOs may be able to conduct genetic risk screening while providing a community of trusted leaders and peers who share culture and language (Almeida et al. 2021; Perez et al. 2023; Vadaparampil et al. 2022). Further, as trusted sources of care and information, CBOs are deeply attuned to the systemic and psychosocial challenges their clients face (Almeida et al. 2021; Perez et al. 2023; Vadaparampil et al. 2022). However, as cancer GCT is still predominantly delivered in specialty (i.e. clinical oncology and genetics) settings (Guan et al. 2021), studies on GCT risk screening via non-specialty (i.e. not clinical oncology and genetics) settings such as CBOs remain nascent (Guan et al. 2021).

A 2021 review by Guan and colleagues (Guan et al. 2021) identified only 16 studies of cancer genetic risk screening in non-specialty settings. They concluded that genetic risk screening was feasible in non-specialty settings, particularly if the screener was brief and integrated into existing processes. Although four of the reviewed studies had significant Latina representation (Lee et al. 2005; Pasick et al. 2016; Anderson et al. 2015; McGuinness et al. 2019), only one reported results separately for Latinas (McGuinness et al. 2019). In this large study of over 3,000 women, of whom 76.7% were Hispanic, only 4.6% of those eligible for BRCA1/2 testing had ever completed testing. The authors identified several barriers particularly relevant to Latinas, including limited awareness of family cancer history, low perceived risk, and missed opportunities for referral by providers. However, they also reported that Hispanic women expressed high interest in genetic counseling and testing when informed, underscoring the importance of culturally tailored outreach.

Several more recent community GCT studies have been published (Amendola et al. 2022; Wu et al. 2022; Wurtmann et al. 2022; Swisher et al. 2020; Nazareth et al. 2021; Conley et al. 2021; Hurtado-de-Mendoza et al. 2020), including a few focused on Latinas (Almeida et al. 2021; Perez et al. 2023; Bowen et al. 2023; Hurtado-de-Mendoza et al. 2020; Hurtado-de‐Mendoza et al. 2021; Tamayo et al. 2022), yet, there remains a need for systematic, implementation science and equity-guided examination of barriers and facilitators to community-based GCT risk screening among the underrepresented population of Latinas.

Existing studies of cancer GCT among Latinas (Ramirez Leon et al. 2024; Dron et al. 2023) rarely use implementation science equity-guided frameworks. Implementation science refers to “the scientific study of methods to promote the systematic uptake of research findings and other evidence-based practices into routine practice” (Eccles and Mittman 2006). Implementation science places special importance on studying contexts where “context” is the delivery setting, priority population, or both (Bauer et al. 2020; Bauer et al. 2015). Thus, implementation science is appropriate for the study of GCT promotion through Latina-serving CBOs. Yet, as many implementation science frameworks have lacked an explicit focus on equity (Adsul et al. 2022; Brownson et al. 2021; Kerkhoff et al. 2022; Snell-Rood et al. 2021; Woodward et al. 2021; Shelton et al. 2021), there have been concerns that implementation research will exacerbate inequities (Brownson et al. 2021; Shelton and Brownson 2024) unless frameworks are adapted to explicitly center health equity, examining the role of structural, socioeconomic, and potentially cultural factors of access.

The purpose of the current work is to address a gap in the science of how to promote access to GCT risk screening among Latinas through CBOs using an implementation science and equity-guided approach. This work is a part of a larger hybrid type 1 effectiveness-implementation trial of GCT delivery in Latina-serving CBOs. Guided by a modified CFIR 1.0 framework, we aim to explore the barriers and facilitators encountered nine months after implementing the HBOC risk screener at four CBOs serving Latinas. Findings from this work can inform future efforts to assess feasibility, acceptability, and sustainability of GCT screening in similar community contexts.

## Methods

The current work was a qualitative study design employing focus groups with CBO staff and a brief follow-up demographic survey. The institutional review board at Georgetown University approved the study protocol.

### Modified CFIR 1.0 framework and theoretical approach

We applied an established implementation science framework, the Consolidated Framework for Implementation Research (CFIR), to guide our qualitative implementation study. CFIR examines barriers and facilitators of implementation with a focus on context (Damschroder et al. 2022a). Thus, CFIR is particularly well suited for studying barriers and facilitators of implementation of cancer genetic risk screening in the context of community-based settings serving Latinas. We employed a modified CFIR 1.0 framework. The modified CFIR 1.0 retains the original five domains (*Process*,* Intervention Characteristics*,* Inner Setting*,* Outer Setting*,* and Characteristics of Individuals*) (Senier et al. 2019) and is supplemented with two elements from CFIR 2.0 (Damschroder et al. 2022a, b): the Adapting construct (under the *Process* domain), as well as an analytic lens oriented toward Health Equity (see Supplementary Table S1 for domain and construct definitions). We added the Adapting construct to identify changes that CBO staff made or recommended making to the screener and/or to the CBO. We added the Health Equity lens to examine equity-related considerations, including social determinants of health, structural barriers, and cultural responsiveness across all domains, in line with our study aims.

### Sampling and recruitment

Staff from our four community-based organization (CBO) partners were eligible to participate in the focus groups. Our CBO partners are from the larger, aforementioned trial, are diverse in their missions, size, and activities, and are longstanding partners who serve Latinas in the Washington DC and Virginia area (See Table 1 for CBO shorthand names and descriptions). Individuals were eligible to participate in focus group discussions if they were full-time, part-time, or volunteer staff at one of the CBOs, were 18 years old or older, were fluent in English or Spanish, and were able to provide informed consent. To recruit participants, we requested CBO leaders to circulate the opportunity to join the focus group with their staff. As a part of the study, the CBO staff participated in a virtual training on HBOC, risk factors, genetic counseling and testing, how to administer the HBOC screener, and the study.

**Table 1:** CBO descriptions

	CBO shorthand	Description
CBO 1	Non-clinical cancer navigation CBO	Focuses on promoting cancer early detection and survivorship care through patient navigation; they support approximately 1,200 Latinas annually
CBO 2	Academic community cancer navigation CBO	Provides cancer education and navigation to cancer screening services for under-resourced populations; serving about 750 women annually ≈ 50% of whom are Latina. Affiliated with an academic hospital.
CBO 3	Latino health promotion CBO	Non-profit organization connecting the Latino community with preventative care services such as diabetes, hypertension, and cancer screening
CBO 4	Free clinic CBO	Provides free healthcare to nearly 1,600 patients annually, 80% of whom are Latine, catering to low-income, uninsured adults.

## The HBOC genetic risk screener

An adapted Family History Screener 7 (FHS-7) (Ashton-Prolla et al. 2009) was implemented at all CBOs. As a part of the larger, aforementioned trial, all four CBO partners previously selected the FHS-7 screener from a menu of five evidence-based screener options and adapted it for cultural responsiveness and consistency with the most recent NCCN guidelines (Bowen et al. 2023). The adapted FHS-7 has 10 yes/no questions. If the answer to any of the items is ‘yes’, this indicates that the individual is at-risk for HBOC, and eligible for genetic counseling referral (Moyer 2014; Ashton-Prolla et al. 2009).

### Study procedures

Four focus groups were conducted virtually from June-August 2021, one for each CBO, with 4–8 staff per CBO for a total of 26 CBO staff participants. Focus groups were organized with the approval from each CBO’s leadership, were 1–1.5 h, conducted in English (*N* = 2) and Spanish (*N* = 2), moderated by the principal investigator (AH) and project coordinator (SG), both Latina bilingual female researchers. A trained study member took notes during each focus group, which were also recorded via Zoom and transcribed. Transcriptions were de-identified. We also administered a brief, electronic demographic survey to collect data on focus group participants’ professional role, the time they had worked at the CBOs, the time they had been in their specific roles, age, education, race, ethnicity, and sex. Participants received $20 as compensation for their participation.

### Focus group guide

Our semi-structured focus group guide (see Supplementary Table S2) was developed according to the modified CFIR 1.0 described above. The focus group guide questions asked about: *Process*, referring to strategies used to implement the screener (e.g., Adapting screener items, identifying who conducts screening, establishing timing) with attention to whether processes accommodated structural or social inequities affecting access. *Intervention Characteristics* refers to perceived relative advantages, complexity, cost, and adaptability of the evidence-based intervention (i.e., the adapted FHS-7), with attention to how these characteristics intersect with cultural responsiveness and address literacy barriers faced by under-resourced populations. *Inner Setting* refers to CBO’s internal environment and infrastructure (e.g., available resources, compatibility), with attention to whether organizational capacity promoted or hindered equitable implementation. *Characteristics & Roles of Individuals* refers to the characteristics, preferences, needs, barriers, and facilitators among CBO staff (e.g., knowledge and beliefs, self-efficacy), with emphasis on how staff capabilities, trust with patients, and shared lived experiences contributed to equitable delivery. While we did not explicitly include questions on *Outer Setting* (i.e. the broader economic, political context in which the inner setting operates), findings on *Outer Setting* partnership with external organizations and their responsiveness to patient’s needs and resources, emerged and were coded as Cosmopolitanism and Patient’s need and resources. To apply the Health Equity lens, our focus group addressed equity-related considerations, including social determinants of health, structural barriers, and cultural responsiveness across all domains.

### Analysis

For quantitative demographic survey data, we applied descriptive statistics to characterize the study sample. For the qualitative focus group data, Spanish transcriptions were translated into English. We employed template analysis (Brooks et al. 2015), a type of thematic analysis using an a priori codebook. The codebook comprised codes for the modified CFIR 1.0 domains and constructs with their definitions. Two research team members coded the transcripts independently (BO, AH) in Dedoose (SocioCultural Research Consultants, LLC 2023), a software application for qualitative analysis. All content was coded under the relevant modified CFIR constructs and as either a barrier or a facilitator. Further, relevant content was coded under Health Equity.

Coding discrepancies and any revisions to the template were solved by consensus, as recommended by the Consensual Qualitative Research Framework (Hill et al. 1997). Subsequent to coding, four research team members (BO, MH, DS, CB) reviewed coding results by CFIR domain and construct and synthesized the results, producing the current manuscript. Rigor was enhanced through peer debriefing and member checking.

### Positionality statement

The authorship team is built from a research-practice partnership between a university and four CBOs. The team is comprised of a primarily Latina and yet diverse group of academic and community-based scholars, including 10 faculty members, 10 CBO administrators and staff, and five undergraduate through postdoctoral researchers. Our team was majority female. Throughout the study, the team actively leveraged the diverse roles and identities within both academic and community contexts. The shared cultural and linguistic backgrounds of the researchers provided nuanced interpretations of data, particularly regarding the Health Equity challenges faced by Latinas. These identities also informed a reflective approach, shaping how the team engaged with participants and influenced the interpretation and reporting of findings.

## Results

As shown in Table 2, CBO staff participants primarily self-identified as Hispanic (76.0%). CBO staff were patient navigators (42.1%), community outreach educators (36.8%), administrative staff (10.5%), and staff in leadership roles (10.5%). Table 3 reports the number of screeners completed. Despite the COVID-19 pandemic, all CBOs demonstrated considerable growth in completed screeners, ranging 38–469 screeners in the post-implementation Jan 2021-Sep 2021 period (total *N* = 789). The lower end of the range is largely due to a late start in July 2021 for CBO three (Latino health promotion CBO).

**Table 2:** Background characteristics of CBO staff focus group participants (*n* = 26^a^)

Characteristic	
Race	
Black/African American	12.0%
White	36.0%
Asian	8.0%
More than one race	16.0%
Other	16.0%
No answer	12.0%
Ethnicity	
Latine	76.0%
Non-Latine	20.0%
Prefer not to answer	4.0%
Staff role ^b^	
Patient Navigators	42.1%
Community Outreach Educators	36.8%
Administrative Staff	10.5%
Leadership Positions	10.5%

^*a*^ One CBO staff participant did not provide this information. Thus, all percentages are for the denominator, *n* = 25

^*b*^ While CBO staff may have multiple roles, we only report the primary role

**Table 3:** Number of genetic risk screeners completed at CBOs across three time periods

Site	# Genetic Risk Screeners Completed		
	9 months prior to the COVID-19 pandemic (Jul 2019-Mar 2020) ^a^	9 months Pre-implementation (Apr 2020- Dec 2020) ^a^	Post-Implementation (Jan 2021-Sep 2021)
CBO 1	5	134	469
CBO 2	0	0	126
CBO 3	0	0	38
CBO 4	0	0	156
*Total*	5	134	789

^*a*^ In time periods before the study, the ‘number of screeners completed’ refers to the number of any standardized family history data collection forms completed. We report numbers for three time periods: (1) Nine months prior to the COVID-19 pandemic, Jul 2019-Mar 2020, (2) Nine months pre-implementation, Apr 2020-Dec 2020, (2) The current work’s nine-month post-implementation period, Jan 2021–Sep 2021. “Implementation” refers to the time *point* at which screeners were first implemented at the CBO (i.e. Jan 2021 for CBOs 1, 2, 4; Jul 2021 for CBO 3). The purpose of reporting data collection in the prior periods is to demonstrate that the number of adapted FHS-7 screeners completed post-implementation is a considerable growth relative to prior periods

Template analysis revealed CBO staff-reported findings on barriers and facilitators by modified CFIR 1.0 domain. In Table 4, we provide key takeaways on barriers and facilitators by domain. We present exemplary quotes from select constructs in Supplementary Table S3.

**Table 4:** Key takeaways on CBO staff-reported barriers and facilitators by modified CFIR 1.0 domain/construct

Domain & Construct	Barriers & Facilitators
Process	
- Adapting	-Barrier: Existing data management system not suitable for genetic risk screening - Facilitator: A standardized, culturally relevant introduction to the screener and item-level adaptations addressing health literacy and cultural responsiveness barriers (e.g. clarify meaning of genetics, Ashkenazi ancestry, triple-negative breast cancer). Use of skip logic to streamline questioning and avoid confusion
Intervention Characteristics	
- Relative Advantage	- Facilitator: Standardization offered by adapted FHS-7
- Cost	- Barrier: Cost burden varied; some CBOs absorbed costs, while cost was barrier for others
- Adaptability	- Facilitator: Easily adaptable screener
-Complexity	- Facilitator: short, simple (y/n) screener
Inner Setting	
- Compatibility	- Facilitator: Aligned with CBO missions on cancer prevention and community engagement; strengthened patient resource connections, relatively easy to integrate in the workflow
- Available Resources	- Barrier: Staff shortages, time constraints, and funding gaps threatened sustainability
Characteristics of Individuals (CBO Staff)	
- Knowledge and Beliefs About the Intervention	- Facilitator: Having a patient navigator serve as the screener administrator, rather than a doctor, was seen as a facilitator. Further, staff valued the implementation of the screener
- Self-Efficacy	- Facilitator: Training and practice boosted staff confidence; additional in-depth training requested.
Outer Setting	
- Cosmopolitanism	- Facilitator: Strong partnerships and level of agency within partnerships aided funding, training, and referrals. -Barrier: Reliance on external institutions (e.g., hospital EHRs) limited implementation.
- Patients needs and resources	- Facilitator: CBOs very aware of patient needs and resources. Expanded access to genetic testing for underserved populations but hindered by low awareness, scheduling difficulties, and access barriers.

### Process

Adapting. Setting up the genetic risk screener involved identifying stakeholders to conduct screening (e.g., navigator, patient self-administration), who will be screened (e.g., all new patients, only those returning for a mammogram), when (e.g., during intake, when returning mammogram results), and changes to the CBO such as updating the data collection systems.

Pre-implementation, only CBO one (non-clinical Latino cancer navigation CBO) systematically collected family history data; two other CBOs (2 and 4) collected some family cancer history data but not systematically, and none followed up on the data collection. Specifically, CBO one (non-clinical Latina cancer navigation CBO) used open-text responses in its electronic software to document non-HBOC-specific family cancer history. CBO two (academic community cancer navigation CBO), collected hereditary cancer-related family history data via an electronic Department of Health intake form with text and checkbox questions about personal and family history of breast and ovarian cancer, including maternal/paternal lineage and age at diagnosis, but neither systematically nor for follow-up. CBO three (Latino health promotion CBO) did not collect any family cancer history data. At CBO four (free clinic CBO), family cancer history data collection varied by provider and when collected was via open-text in their paper intake form.

Post-implementation, two of the three CBOs that had an intake form integrated the screener into their intake. CBO one (non-clinical Latino cancer navigation CBO) integrated the screener into a new electronic intake form, where navigators completed it with all new and returning patients during phone intakes or on-site visits. For in-person visits, navigators completed the screener on paper and then entered the data into the electronic screener which automatically sent an email to a CBO staff when there was a positive result; who then called back the patient to confirm the data. CBO two (academic community cancer navigation CBO) integrated the screener into their electronic REDCap intake form. Before entering data into their intake, however, navigators completed the screener via Google Forms while on the phone with new and returning patients. Google Form data was then transferred to the REDCap intake and Ripple case management systems. Positive results could be searched in Ripple, though this was found to be “cumbersome” and was rarely done. Since CBO three (Latino health promotion CBO) had no existing intake form, navigators completed a standalone Google Form adapted FHS-7 screener while on the phone or in-person with new patients. Positive results were recorded by navigators in an Excel tracker (developed by our study team). Although CBO four (free clinic CBO) had an EHR intake, the CBO was unable to integrate the screener due to lack of agency over the EHR, which was owned by their clinical partner. CBO four’s (free clinic CBO) lack of agency over their EHR was a Health Equity barrier given their patient population is primarily low-income and under/uninsured individuals. The clinic’s strategy to address this barrier was to complete the screener on paper while on the phone with patients, and, then, record only the necessary information (i.e. date of completion and any positive result) in their EHR later. Only one of the CBOs had an automatic flag for positive results. Among the other CBOs, CBO staff reported:

*“It’s not flagged that someone has answered yes. It’s something I see as I go back and I reviewed the Google forms*,* then I’ll notice it. Unless somebody has told me that they have a person who’s positive.” —*CBO two (academic community cancer navigation CBO).

### Intervention characteristics

Relative Advantage*.* The adapted FHS-7 screener was unanimously praised over previous cancer history data collection. The standardization offered by the adapted FHS-7 was particularly valued by CBO staff given systematic varying levels of expertise in collection of family history among CBO staff and medical providers. In terms of Health Equity, CBO staff found that the FHS-7 screener promotes health equity more than prior screening systems:

*“I think we’re appreciative that at least they can have the opportunity or the option of this [genetic counseling and testing]. Because otherwise*,* there would be no way that they could have genetic testing…It’s nice when our patients get the same level of care that insured people get.“—*CBO four (free clinic CBO).

Cost. Intervention-level barriers related to cost were also noted, including the additional time needed to administer the screener (see *Inner Setting*), and the costs to integrate the screener into the CBOs’ database. Cost was a lesser concern for the academic community cancer navigation CBO where costs were minimal and already included in their operational costs, whereas it was a bigger concern for the other CBOs. For example, one CBO described how their ongoing infrastructure transition allowed them to absorb costs more easily:

*“Because we had an intake form*,* the only cost associated*,* because it’s not a big form*,* the cost associated was the programming costs into the REDCap. So*,* that wasn’t a big*,* we were changing over to REDCap while we were working on this form anyway.”*—CBO two (academic community cancer navigation CBO).

Other CBOs identified additional software and time-related expenses as barriers, especially without dedicated funding (see *Inner Setting*).

Complexity. Although CBO staff highlighted that some words in the screener may be difficult to understand (see *Outer Setting*); they appreciated the screener’s simple short list of questions with a yes or no format:

“*It is a simple questionnaire… I find it easy and simple.”*—CBO three (Latino health promotion CBO).

Adaptability. CBO staff praised the screener’s adaptability because its adaptability facilitated addressing the Health Equity literacy, linguistic and/or cultural needs of the population (see *Outer Setting*). For example, to address health literacy barriers the adaptations included adding a short introduction to the screener to explain why collecting this information is important and to link it to other health issues that can run in families and well-known cases (e.g. Angelina Jolie) that patients may be more familiar with.

*“You know when you go to the doctor and they ask you if you have diabetes*, *cholesterol*,* and then they go*,* ‘Oh yeah*,* I remember that.’ That’s the same thing with genetic things. We just wanted to know whether you have someone in your family who might be high risk. It’s like a tree and it’s like your mom and your dad*,* and then your brothers and sisters*,* and then your cousins and your uncle and they were just telling me*,* and then that’s why I have questions because my community*,* the population that I navigate*,* which is Hispanic*,* unfortunately*,* most of them*,* when I was doing the screening*,* they never went to school”* —CBO two (academic community cancer navigation CBO).

Yet, challenges remained when patients had difficulty identifying and categorizing family relationships, which could delay completion and require clinical follow-up:

*“I will say maybe it is a little challenging if people are unsure about the degree of family relations. That causes some challenges and a timely completion*,* but that’s often referred to the provider to help parse out.”* —CBO two (academic community cancer navigation CBO).

### Inner setting

Compatibility. CBO staff reported that cancer genetic risk screening aligned with the CBOs’ missions and values on comprehensive prevention care, cancer prevention, and/or health disparities. One CBO staff explained:

*“Part of [CBO 1]’s mission is to give you access to the community*,* to cancer treatments. But I see cancer treatments a little broader… I see is that the screener is very akin to our mission*,* because it also allows us to provide the service that if these people are saying yes [to screener questions] and we refer them to you*,* maybe one of these people can be saved.*”—CBO one (non-clinical Latina cancer navigation CBO).

Available Resources. Lack of budget was the most prominent barrier to screening. A CBO staff explained that although cancer genetic risk screening was a priority a few years earlier, lack of funds for staffing prevented the CBO from initiating screening:

*“… It was identified a few years ago as one of the big priorities for our catchment area. So*,* it has been a priority for us*,* but we needed to find funding and also support to do that.”* –CBO two (academic community cancer navigation CBO).

Given our CBOs predominantly serve under-resourced populations of Latinas, the CBOs’ lack of funding creates Health Equity barriers to accessing screening. Lack of budget was synonymous with lack of staff time for genetic risk screening:

*“I can tell you that from a management standpoint*,* obviously the disadvantage is time. Time that translates into money.”—* CBO one (non-clinical Latina cancer navigation CBO).

On average, screener administration time was 5–10 min. However, there was a considerable range (see *Patient Needs and Resources)*. In response to the need for staff time for screening, CBO staff reported that sustainability could be challenging:

“*To try and make a goal of having a certain portion of our population be screened once a year … at least once would be feasible if we did it in a different kind of approach. It might not be through primary care. It might be through navigators (…) ” —* CBO four (free clinic).

### Characteristics of individuals (CBO staff)

In terms of Health Equity, patient navigators serving as the screener administrator, rather than a doctor, was seen as a facilitator. Patients experienced greater trust, less perceived hierarchical barriers between patient and administrator, and more open dialogue and effective family cancer history data collection with patient navigators than with other CBO staff, contributing to greater equity in screening delivery. CBO staff conveyed:

“*They feel like she’s not a doctor*,* and she’s not a clinical. She’s just a navigator. So*,* that was very helpful for me to learn. And then in the end*,* I feel like I’m a little prepared to just talk to them in the future.*”—CBO two (academic community cancer navigation CBO).

Knowledge and Beliefs about the Innovation*.* CBO staff reported positive opinions of the screener and its effectiveness for promoting improved health of their patients. A CBO staff stated:

*“We already provide services for our women. So this just completes the whole picture…Knowing that we are a nonprofit and we see uninsured patients*,* I think that this is just a great service to add onto the things that we already do.” —* CBO three (Latino health promotion CBO).

Self-efficacy *.* CBO staff’s strong sense of self-efficacy in implementing the screener was a key facilitator. CBO staff explained that practice led to increased self-efficacy in screening despite initial challenges with CBO staff understanding the purpose and potential pitfalls of the screener:

*“But now we are more prepared*,* and we explain things well and it is no longer complicated for us*,* which at first was like*,* “Oh God*,* how do I say this?”*—CBO one **(**non-clinical Latina cancer navigation CBO).

CBO staff expounded that training, including more in-depth and refresher trainings, were also facilitators:

*“The training so far has been very excellent*,* and we have understood it*,* and we have been able to convey it to the clients. But if there was something more in-depth*,* it would be good for us*,* since learning helps us a lot…” –*CBO three (Latino health promotion CBO).

### Outer setting

*Cosmopolitanism.* The strength of a CBO’s system of external partnerships facilitated screening at the CBOs. Of the four CBOs, the two that had greater external partnerships—CBO one and two—had an easier time implementing genetic risk screening, largely due to stronger infrastructure afforded by the partnerships. As a CBO two staff explained, their ability to administer genetic risk screening was aided by two external partnerships that funded their GCT screening efforts:

*“There is another study that is going on with the Breast Health Equities Study*,* which is a quality improvement implementation study. And that is kind of covering some of the navigated time on to navigate genetic counseling and referral. And it also is providing some funding for infrastructure*,* for setting up*,* for example*,* referral processes*,* and also building new partnerships.”* –CBO two (academic community cancer navigation CBO).

Partnerships with academic hospitals and/or genetics clinics also facilitated ease of referrals to GCT programs, EHR use, and connections to receiving staff training on GCT. Further, a key factor in facilitating or hindering screening was not just the presence of these partnerships, but the level of agency CBOs had within these partnerships. For instance, one CBO with an existing partnership with a large academic hospital struggled with implementation due to its lack of control over the hospital-owned EHR. As a CBO staff explained:

*“We’re actually reliant on [hospital name redacted] to integrate us into their EHR. So it’s kind of like beggars can’t be choosers.”—*CBO four (free clinic CBO).

Patient needs and resources. CBOs had a high awareness of patients’ needs and the barriers and facilitators to respond to said needs, all of which were closely related to Health Equity concerns. At the outset, CBO staff expressed that screening addressed an unmet need and promoted Health Equity as, prior to the screener, individuals outside of breast cancer treatment plans rarely had access to GCT:

*“Unless you were diagnosed with breast cancer and it was a part of your treatment plan to meet with a counselor*,* you weren’t offered this service.”* — CBO two (academic community cancer navigation CBO).

However, CBOs highlighted that one of the patient needs was to call outside of business hours to conduct the risk screening. Finding a mutually convenient time for patients and CBO staff to conduct the screener phone calls was challenging given that both parties often have multiple jobs and responsibilities. Thus, CBO staff identified that a facilitator was to call patients during lunch, on weekdays after business hours or over the weekend.

*“People aren’t there – or maybe they don’t have the time. But we try to ask them what the best time to call is… But yes*,* I think personally it’s been that*,* getting people to answer the phone –*CBO three (Latino health promotion CBO).

CBO staff also reported health literacy barriers with medical jargon among patients:

*“Because of language and wording and literacy level and health literacy level and all those things*,* what seems like a simple question if you ask me*,* “Have two more families had breast cancer*,* ovarian cancer” I could say to you in a second yes or no. Whereas*,* like [redacted] was explaining before*,* for her that could be a several-minute conversation.”—*CBO four (free clinic CBO).

To address health literacy barriers, CBO staff recommended provision of a *“cultural competency-based standardized paragraph”* explaining hereditary cancers and why collecting family history information is important. Beyond the introduction, CBO staff recommended item-specific adaptations to clarify jargon, addressing health literacy barriers. Patients had difficulty understanding triple negative breast cancer, differentiating ovarian cancer from other gynecological cancers, tumor vs. germline testing, and they were unfamiliar with Ashkenazi Jewish ancestry:

*“A specific question that often creates confusion*,* is when I ask about if you ever have been diagnosed with triple negative breast cancer… They tend to say ‘Yes*,* yes*,* negative*,* negative*,*’ [meaning they are negative for breast cancer]. So*,* I adapt and clarify by explaining triple negative is a specific type of cancer we’re referring to.” –*CBO one (non-clinical Latina cancer navigation CBO).

Likewise, CBO staff advocated for allocating more than the expected 5–10-minute session for screening. CBO staff explained Latina patients may need additional time to gather comprehensive family cancer history information, as this information is not always discussed among families and they often had to call relatives living outside the country, thus, crossing geographic barriers to collect family history information.

Allowing time for patients to collect this information was particularly important as “*one of the challenges is that*,* that the information is not the same from the beginning to the end”.* Specifically, in the process of speaking to more relatives, the details become more accurate, as explained by a CBO staff:

*“It’s very interesting that sometimes the information they give to the girls is not the same as the information they give to me. Because when they talk [redacted] for the first time*,* they tell them who in their family. When I call them to confirm the information*,* they probably tell me the same thing*,* but they add one more person*,* or there is something different. And when they do the interview with the genetic counselor*,* it turns out that it was not cancer*,* …Or it turns out that the mother*,* instead of having had cancer – she told me at 48*,* she told the genetic counselor at 70.” –*CBO one (non-clinical Latina cancer navigation CBO).

The lack of accurate family cancer history information may also stem from cultural norm variations in perceptions and access to preventive care, as relayed by a CBO staff:

*“To come from countries where healthcare – no one goes into the hospital except when they die. You know? So it’s a little bit of a needle in a haystack trying to figure out what actually happened.” —*CBO four (free clinic CBO).

Incomplete family cancer history information may also be due to viewing disclosure of cancer diagnoses as “taboo”, as “*many families do not like to say that they have cancer. Sometimes they hide it and do not say anything”* (CBO one; non-clinical Latina cancer navigation CBO). Variations in defining “family” may also contribute to inaccurate family cancer history collection. A CBO staff recounted a scenario where a relative referred to as “aunt” turned out to be a more distant relative and she ended up not being recommended for genetic testing when she attended genetic counseling.

*“[In] the Latino community…we say*,* ‘My aunt had cancer. And breast cancer before the age of 50.‘… But it turns out that the aunt was not her real aunt*,* but that she was the mother’s cousin*,* but she calls her aunt.” –*CBO one (non-clinical Latina cancer navigation CBO).

CBOs also discussed how implementing the screener often took longer since Latinas also tended to share their or relatives’ social determinants of health or other health concerns with the navigators:

*“One of the things is that obviously when they talk to the community*,* not only do they bring up the problems they may have related to health*,* but they are also talking to you about the problem they have of not being able to pay a bill or that their child cannot go to school. They confess to you and then it is very difficult to stop them*,* right?” –*CBO one (non-clinical Latina cancer navigation CBO).

CBOs shared that gathering cancer family history information can also trigger emotional reactions that need to be addressed:

*“And if she has cancer*,* she starts crying*,* or if her cousin or her sister. And they tell you the story. And you are there to listen to them and be able to offer support. You can’t tell them*,* “No ma’am*,* calm down*,* I’m not talking to you for that. Just for a mammogram.”—* CBO one (non-clinical Latina cancer navigation CBO).

And as explained by the CBO staff, many of these conversations are aided by the trust felt with navigators to explain life issues. This may also be a conversational style among Latinas, according to CBO staff:

*“You see that the Americans tell you*,* “Tell me about the color white.” And the person only talks about the color white. But to the Latino you say*,* “Tell me about the color white.” And then they mix up red*,* yellow*,* green*,* and they don’t even talk about white. So what is happening with the other [CBO staff names redacted] is that at some point the ladies start to tell or ask for help with other needs.” –*CBO one (non-clinical Latina cancer navigation CBO).

CBO staff found they had to incorporate time to research referral pathways and complete associated steps (e.g. obtaining prior authorization for coverage) to ensure both insured and uninsured patients were equitably receiving access to risk screening:

*“I just*,* I’m a little bit weary of continuously referring insured patients to testing*,* not knowing if their insurance will cover it.”* — CBO two (academic community cancer navigation CBO).

CBO staff recommended developing a CBO-facing implementation toolkit:

*“Like if there was a recommendation for a patient navigation kind of genetic counseling toolkit or something basic course that every year we could go back to*,* that every year the patient navigator could go back to and say*,* “This is what this is. This is what we ask.” –*CBO two (academic community cancer navigation CBO).

The purpose of the toolkit would be to compile implementation insights, such as “*what kind of words and phrases*” to use and why and “*resources so where they can go*”, according to CBO two (academic community cancer navigation CBO) and CBO three (Latino health promotion CBO) staff, respectively.

## Discussion

The current work aimed to systematically examine the barriers and facilitators to implementing cancer genetic risk screening among community-based organizations (CBOs) serving Latinas in the Washington DC-Virginia area. CBOs successfully implemented screening, administering 789 screeners across nine months during COVID-19. Employing a modified CFIR 1.0 framework, we identified barriers and facilitators for cancer genetic risk screening to address. Our findings may be transferable to knowledge on promoting equitable access to health screeners.

Our work identifies a *Process* barrier to GCT risk screening in CBO settings that has rarely been highlighted—setting up an optimal data management system for screening. Although nine studies in Guan and colleagues’ 2021 systematic review of GCT in non-specialty studies appeared to have an EHR, only two integrated the screener into the EHR. EHR integration was not explicitly identified as a barrier in the systematic review or the underlying studies. Yet, given the low proportion of settings that integrated the screener into their EHR, EHR integration appears to be an ongoing barrier in non-specialty settings warranting future research. Further, there is a need to update data management systems that are compatible with genetic risk screening implementation. Our identified need to update data management systems aligns with recent findings that EHR integration of genetic risk screeners may also be a GCT barrier in clinical *specialty* settings (Lau-Min et al. 2022; Wang et al. 2023). In specialty settings, electronic health record (EHR) systems lack structured integration for GCT data, requiring re-design. Cancer screening, in particular, can be hindered by EHR challenges (Janett and Peter 2020; Berger et al. 2016; Tayefi et al. 2021).

This study highlights important strategies to adapt GCT risk screening for Latinas. Specifically, our CBO staff recommended a brief introduction and item-level changes to the screener addressing health literacy and cultural responsiveness. While prior studies of GCT among Latinas (Almeida et al. 2021; Perez et al. 2023; Hurtado-de-Mendoza et al. 2020; Hurtado-de‐Mendoza et al. 2021; Tamayo et al. 2022) including our study of pre-implementation adaptations (Bowen et al. 2023) have identified key adaptations, there were notable adaptations that our CBOs identified post-implementation through the current work (e.g. triple-negative breast cancer).

The recommendation from our CBO staff for a CBO-facing GCT implementation toolkit with information on local resources is consistent with evidence from the implementation science field. The established Consolidated Framework for Implementation Research–Expert Recommendations for Implementing Change (CFIR-ERIC) Matching Tool (Waltz et al. 2019) was developed to help researchers and practitioners understand which ERIC implementation strategies will best address which CFIR barriers. In response to several barriers (e.g. *Inner Setting— Available Resources* (e.g. lack of staff time) barrier), a commonly recommended ERIC implementation strategy has been to ‘capture and share local knowledge’ (Waltz et al. 2019). Thus both our current findings and the existing literature are consistent in their recommendation to develop an implementation and local resources toolkit to promote screening uptake at CBOs, and potentially other health screeners in diverse contexts. Future studies should further examine potential implementation strategies to overcome barriers and leverage facilitators across all the domains.

As related to *Characteristics of Individuals*, focus group discussions highlighted that patient navigators were more effective than medical providers in conducting screenings for the under-resourced Latina population due to their earned trust and shared language and culture with the community. During screening, patients frequently shared other emotional, health, or broader concerns. This highlights that in this population, screening is embedded within social determinants of health needs that must also be addressed. While “low-touch” or less time-intensive approaches to screening may be feasible in some settings, further studies should assess whether such approaches are appropriate and equity-promoting for underserved minority populations.

### Limitations and strengths

Key limitations of this work must be considered. One limitation relates to the transferability of findings to other CBO settings. This study covered the costs of genetic counseling through the study grant, and the research team managed most referrals, including assistance with financial aid applications for genetic testing. Therefore, we did not examine barriers and facilitators related to patient navigation through the full GCT continuum. However, this study provides a nuanced description of the CBOs’ experiences implementing genetic risk screening in real-world settings. Future studies should address all phases of the continuum, including counseling, testing, and follow-up risk management.

A second limitation involves the generalizability of findings beyond the Washington DC–Virginia region. CBOs serving different Latina subpopulations or rural communities may require distinct adaptations. Additionally, although we assessed the type of family cancer history data collected at each CBO before enrollment, we did not have data on the number of completed GCT referrals prior to our study, limiting our ability to evaluate changes over time.

Finally, while this study used the CFIR framework to identify barriers and facilitators to implementation, CFIR 1.0 does not directly assess implementation outcomes such as feasibility or acceptability. Some constructs such as Available Resources (level of resources dedicated for implementation and ongoing operations), Knowledge and Beliefs (attitudes toward and value placed on the innovation), and Compatibility (the degree of fit between the innovation and the organization’s norms, values, and existing workflows and systems) may indirectly inform perceptions of feasibility and acceptability. However, we did not use formal implementation outcome measures. As such, the findings offer preliminary insight into the implementation outcomes of perceived feasibility and acceptability, albeit without direct measurement. It is possible to interpret CBO staffs’ experiences as exploratory findings that genetic risk screening is feasible and acceptable across the sampled CBOs once barriers and facilitators are addressed. Future research should include validated instruments, such as those described by Proctor et al. (2011) to directly assess perceived feasibility, acceptability, and appropriateness in community-based settings.

Despite these limitations, this study provides a nuanced account of how CBOs implemented genetic risk screening in real-world contexts. It advances the science of equitable access to cancer GCT, particularly for Latinas, and contributes meaningfully to the limited evidence base on implementation science in translational genomics.

## Conclusions

While initial studies have suggested that implementing a short, adaptable screener protocol at CBOs may be promising, the evidence base on community-based GCT service delivery remains nascent. The current work study provides foundational insight into the contextual barriers and facilitators that shape the implementation of genetic risk screening in Latina-serving CBOs. These findings indicate that implementation may be perceived as feasible and acceptable when multilevel barriers are addressed and facilitators leveraged. Future work should build on these findings by measuring feasibility and acceptability using validated implementation outcome tools to better guide sustainable and equitable adoption.

## Supplementary Information

Below is the link to the electronic supplementary material.

Supplementary Material 1 (DOCX 20.0 KB)

Supplementary Material 2 (DOCX 23.4 KB)

Supplementary Material 3 (DOCX 24.4 KB)

## Data Availability

The authors welcome inquiries from investigators interested in possible collaboration and use of de-identified data from this study. The data has not been placed into a public repository.
