# Supplementary material for: Barriers and facilitators of cancer genetic risk screening at community-based organizations serving Latinas
Source: J Community Genet. 2025 Dec 16;17(1):16. doi: 10.1007/s12687-025-00839-7 (PMC12708480; doi:10.1007/s12687-025-00839-7)
Supplement: Supplementary file 1 — Supplementary Material 1 (DOCX 20.0 KB) [file 12687_2025_839_MOESM1_ESM.docx]

**Table S1**

**Health Equity-Integrated Definitions of Modified CFIR 1.0 Domains and Constructs**

| **Domain** | **Construct** | **Definition (with Health Equity Lens)** |
| --- | --- | --- |
| *Process* |  | The strategies used to implement the screener (e.g., Adapting screener questions, identifying who conducts screening, and establishing timing), analyzed with attention to whether processes accommodated structural and/or social inequities affecting access. |
|  | Adapting | Changes that CBO staff made or recommended making to the FHS-7 screener, and/or to the CBO to promote greater health equity between the intervention and the context, and greater equity. |
|  |  |  |
| *Intervention Characteristics* |  | The perceived relative advantages, complexity, cost, and adaptability of the evidence-based intervention (i.e., the adapted FHS-7), with particular attention to how these characteristics intersect with cultural responsiveness and address literacy barriers faced by under-resourced populations. |
|  | Relative Advantage | CBO stakeholders’ perceptions of the advantage of implementing the FHS-7 screener versus an alternative solution, particularly to promote health equity. |
|  | Cost | Costs of the FHS-7 screener and costs of implementing the FHS-7 screener including investment, supply, and opportunity costs, with particular attention to inequitable cost burden. |
|  | Adaptability | The degree to which the FHS-7 screener can be adapted, tailored, refined, or reinvented to promote equity. |
|  | Complexity | Perceived difficulty of implementation, reflected by duration, scope, radicalness, disruptiveness, centrality, and intricacy and number of steps required to implement. |
| *Inner Setting* |  | The CBO’s internal environment and infrastructure (e.g., available resources, compatibility), with attention to whether organizational readiness and capacity promoted or hindered equitable implementation. |
|  | Available Resources | The level of resources dedicated for implementation and on-going operations, including money, training, education, physical space, and time, with particular attention to inequities in resource access. |
|  | Compatibility | The degree of tangible fit between meaning and values attached to the intervention by involved individuals, how those align with individuals’ own norms, values, and perceived risks and needs, and how the intervention fits with existing workflows and systems. |
|  |  |  |
|  |  |  |
| *Outer Setting* |  | The broader economic, political, and social context in which the inner setting (i.e., the CBO) operates, including partnerships with external organizations and their responsiveness to patients' needs and resources, particularly social determinants of health impacting Latinas. |
|  | Cosmopolitanism | The degree to which a CBO is networked with other external organizations. |
|  | Patient Needs & Resources | The extent to which patient needs, as well as barriers and facilitators to meet those needs, are accurately known and prioritized by the organization, and how equitably. |
|  |  |  |
| *Characteristics & Roles of Individuals* |  | The characteristics, preferences, needs, barriers, and facilitators among CBO staff (e.g., self-efficacy, knowledge and beliefs), with emphasis on how staff capabilities, trust with patients, and shared lived experiences contributed to equitable delivery. |
|  | Knowledge & Beliefs about the Intervention | Individuals’ attitudes toward and value placed on the intervention as well as familiarity with facts, truths, and principles related to the intervention. |
|  | Self-Efficacy | Individual belief in their own capabilities to execute courses of action to achieve implementation goals. |
|  |  |  |
