# Supplementary material for: Barriers and facilitators of cancer genetic risk screening at community-based organizations serving Latinas
Source: J Community Genet. 2025 Dec 16;17(1):16. doi: 10.1007/s12687-025-00839-7 (PMC12708480; doi:10.1007/s12687-025-00839-7)
Supplement: Supplementary file 2 — Supplementary Material 2 (DOCX 23.4 KB) [file 12687_2025_839_MOESM2_ESM.docx]

**Implementation Needs Assessment Focus Group Guide- Session 2**

**Introduction**

Thank you so much for your participation in this focus group. The goal of this focus group is to discuss your experiences using the short hereditary breast and ovarian cancer (HBOC) screener, that we selected a little over a year ago and implemented at your institution.

**IMPLEMENTATION PROCESS**

**After our last focus group, we selected a screener to implement in your organization which was then used to identify potentially eligible participants for our study. We want to learn about how implementing the screener went for you.**

1. How did you implement the HBOC screener within your organization?
   1. *Prompt:* Was the format electronic/paper-pencil.
      1. **If electronic,** what did that look like? How was that process of integrating it? Were there any costs? How long did it take? Did you need any formal approval?
   2. Who was in charge of administering the screener?
      1. For those who administered the screener, could you walk us through the process (context, how it was introduced to patients, steps before and after administering the screener)
   3. How was the information gathered? (e.g., in person, over phone, online)
2. How was the screener integrated within your clinical practice?
   1. To which patients/clients did you administer it? (e.g., all new patients, existing patients, certain patients)
   2. When was it administered? (e.g., intake forms)
   3. How was the information stored or entered into the system?
   4. How did you retrieve the information about those who screened positive?
3. How was the experience of implementing the screener within your organization?
   1. Any barriers/challenges?
   2. Any things that helped?
   3. Things that could be improved

**Now, we would like to discuss different aspects of the screener, your organization, and the staff involved to understand the things that may been challenging as well as the things that may have helped to implement the screener and referral process.**

**Now we will talk about different aspects of the screener**

**Intervention Characteristics**

###### Relative Advantage

1. How does the use of the short screener compare to prior ways of screening for hereditary cancers in your organization?
   - What advantages does the screener used have compared to your prior ways of screening?
   - What disadvantages does the screener used have compared to your prior way of screening?

*Adaptations*

- 1. Did you make any adaptations to the screener while implementing it?
  2. Are there any additional changes or alterations that you think you will need to make to the screener to continue using the screener?

###### Complexity

1. How complicated was the screener to implement within your setting?
2. How complicated was the screener to administer to patients/clients?
   - Length:
     1. What did you think about the length of the screener?
     2. How long did it usually take you to do one screener? How long did it take you to record the answers? How long did it take you to figure out if the person should be referred to the study or not? How long did it take you to refer them to the study?
     3. Is that length appropriate? What would be the ideal length or time-commitment per patient?
   - Steps:
     1. How difficult was it to follow the steps: administer the screening, record into the system, identify and refer potentially eligible participants?
   - Scope:
     1. Were the questions in the screener ok? Are there are any missing questions or any questions that you found unnecessary or challenging for patients to answer?

**Now we will talk about different aspects of the clinic/ CBO that may have been helpful or challenging to implement the screener**

**Inner Setting**

###### Goals & Feedback

1. How did the implementation of the HBOC screener aligned with other organizational goals?

###### Implementation Climate

1. What was the general level of receptivity in your organization to using the screener?
   - Why?

###### Relative Priority

1. How important do you think it was to implement the screener compared to the other priorities at your organization?

###### Compatibility

1. How well did the screener fit with existing work processes and practices in your setting?
   - Did you find any complications or barriers when using the screener?
2. How well was the screener integrated into current processes and practices?

*Available Resources*

1. Did your organization have sufficient resources to implement the screener?
   - [If Yes] Which resources did you use?
   - Are there any other resources that you received, or would have liked to receive?

**Now we will talk about different aspects and perceptions from the staff involved in the implementation of the screener**

**Characteristics of Individuals**

###### Knowledge & Beliefs about the Intervention

1. Do you think the HBOC screener was effective in your setting?
   - Why or why not?

###### Self-efficacy

1. How confident did you feel when implementing the HBOC screener?
   - What gave you that level of confidence (or lack of confidence)?
   - What could be helpful to increase the level of confidence?

Ok. We are almost done with time and I have asked all the questions I had for you. Is there anything else that anyone wants to bring up or mention that we did not discuss already?

Thank you very much for your participation!

[End recording]
