# Supplementary material for: Barriers and facilitators of cancer genetic risk screening at community-based organizations serving Latinas
Source: J Community Genet. 2025 Dec 16;17(1):16. doi: 10.1007/s12687-025-00839-7 (PMC12708480; doi:10.1007/s12687-025-00839-7)
Supplement: Supplementary file 3 — Supplementary Material 3 (DOCX 24.4 KB) [file 12687_2025_839_MOESM3_ESM.docx]

**Supplementary Table S3**

**Exemplary quotes from select constructs by modified CFIR 1.0 domain/construct**

| **CFIR Domain** | **Definition** | **Quotes** |
| --- | --- | --- |
|  |  |  |
| Process:  Adapting | Changes that CBO staff made or recommended making to the FHS-7 screener, and/or to the CBO to promote greater health equity between the intervention and the context, and greater equity. | Regarding including the screener questions into their intake system: “It looks great, because that way we don't have to call the person again and ask those questions. And since it is part of our intake, practically for us the basic questions were already asked, and until now we have not had any refusal from the clients” –CBO one (non-clinical Latina cancer navigation CBO) |
| Intervention Characteristics:  Relative Advantage  Costs | CBO stakeholders’ perceptions of the advantage of implementing the FHS-7 screener versus an alternative solution, particularly to promote health equity.  Costs of the innovation and costs associated with implementing the innovation including investment, supply, and opportunity costs. | “A lot of our doctors are – well, they're all volunteers. And some of them... may be retired, or they may actually be an emergency room doctor and not a primary care doctor. So anything that standardizes practices is really good for us.”—CBO four (free clinic CBO)  "The costs involved in going forward are obviously the REDCap costs, but also the costs included with the Ripple software that we use to manage participants and make sure we’re following participants after the genetic counseling and testing, and if it’s needed for additional referral. So, some of that cost is again, kind of built into our operations."—CBO two (academic community cancer navigation CBO) |
| Complexity  Adaptability | Perceived difficulty of the innovation, reflected by duration, scope, radicalness, disruptiveness, centrality, and intricacy and number of steps required to implement.  The degree to which the FHS-7 screener can be adapted, tailored, refined, or reinvented to promote equity. | “It's a pretty minimal ten additional questions on a 60-question intake form.”—CBO two (academic community cancer navigation CBO)  “The issue of not asking one by one, but asking directly, ‘Have you had any family members with cancer? who and at what age?’…If the person has told me, ‘No, I don’t have any family members with cancer.’ We know that in all of them we are going to put, ‘No, no, no.’…to make it quicker and flow faster, we kind of base it on those three questions.”—CBO two (academic community cancer navigation CBO) |
| Inner setting:  Compatibility | The degree of tangible fit between meaning and values attached to the innovation by involved individuals, how those align with individuals’ own norms, values, and perceived risks and needs, and how the innovation fits with existing workflows and systems. | “So, I think that including these genetic counseling questions into our intake form has been really helpful with us just fulfilling that new goal of expanding beyond breast cancer screening and engaging the community to get counseling and testing for genetics”—CBO two (academic community cancer navigation CBO) |
| Available resources | The level of resources organizational dedicated for implementation and on-going operations including physical space and time. | “Extra questions for us take time, effort, and money. Because time for us is obviously money. So every time we make a change in the intakes that we do, we also have to evaluate …. So that is more expensive.”—CBO one (non-clinical Latina cancer navigation CBO) |
| Characteristics of individuals:  Knowledge and beliefs about the intervention | Individuals’ attitudes toward and value placed on the innovation, as well as familiarity with facts, truths, and principles related to the innovation. | “The benefit of speaking with women about genetics is educational for them. It seems very beneficial because it makes people think, especially since many of these individuals don’t know their family’s health history…At a community level, I find this very advantageous.”—CBO one (non-clinical Latina cancer navigation CBO) |
| Self-efficacy | Individual belief in their own capabilities to execute courses of action to achieve implementation goals. | “I feel really comfortable during the screening because I liked the fact that it has a script. You have two paragraphs that are very clear that you can read… I think that the paragraphs do a good deal of explaining and answering their questions about why you are asking this set of questions. My experience is it has been good and I feel comfortable administering the screener.”—CBO two (academic community cancer navigation CBO)    “I feel good asking the questions. I think that most of all it is the way you talk to the lady and the confidence that is given to her, that they feel safe, and let them know that everything we talk about is confidential, that it is between her and me, that it is not pass this information on to anyone else, that everything is safe. You have to provide them with security before everything else. But yeah, I feel good doing it.”—CBO one (non-clinical Latina cancer navigation CBO) |
| Outer setting:  Patient’s needs and resources of those served by the organization | The extent to which the needs of those served by the organization (e.g., patients), as well as barriers and facilitators to meet those needs, are accurately known and prioritized by the organization. | “Lately, what was working better for me was calling on Saturday, because there was a better chance of finding them. Because I can't even say I leave a message and they call me back because they usually don't listen to messages.”—CBO one (non-clinical Latina cancer navigation CBO) |
|  |  | “It could also have to do with the Latin American taboos within families. For example, in my family, there are many people who have had different types of cancer, and I only recently found out. Many families don’t like to admit they have cancer. Sometimes they hide it and don’t say anything, or if they do say someone is sick, they admit it later, and say 'Yes, it was cancer.' When they told the family it was maybe a cold.”—CBO one (non-clinical Latina cancer navigation CBO)  “It is interesting to know...and we already know because we've been working with this population for many years at this point. But there are people who don't know how their closest relatives have passed away. So that's a biggie one. They just know a big idea that it was probably cancer.”—CBO four (free clinic CBO)  “A patient told me that they do not want to know their results due to fear the patient told me that her brothers and sisters and a lot of family members died of cancer. So, they’re very scared and just want to enjoy their life, and then just don’t want to think about that.”—CBO two (academic community cancer navigation CBO |
| Cosmopolitanism | The degree to which an organization is networked with other external organizations. | "The good thing about these genetics programs is that we have been working with you and with other organizations, it is that we started little by little, little by little. It was not something that we had a great program, that CBO one could not develop experience, but rather pilot studies, for example, and we left, as they say in English, getting our feet wet little by little. So now we feel that we are at another level.”—CBO one (non-clinical Latina cancer navigation CBO)  “Because since [CBO name] is like the organization to publicize the resources that exist for the community, then this is one of them. And I think so, apart from how I mentioned at the beginning as well, I think it's been beneficial too, because given that, maybe if someone doesn't qualify for the study. But we tell them where we are calling from, from the organization. Then they are getting to know each other as well. If they did not know about the organization, then they get to know, because they pass the information on to other people. So, I think it has been beneficial.”–CBO three (Latino health promotion CBO) |
|  |  |  |
|  |  |  |
